# Emulsification mechanism in an ultrasonic microreactor: Influence of surface roughness and ultrasound frequency

**DOI:** 10.1016/j.ultsonch.2023.106323

**Published:** 2023-02-08

**Authors:** Aniket Pradip Udepurkar, Christian Clasen, Simon Kuhn

**Affiliations:** a Department of Chemical Engineering, Process Engineering for Sustainable Systems (ProcESS), KU Leuven, Celestijnenlaan 200F, 3001 Leuven, Belgium; b Department of Chemical Engineering, Soft Matter, Rheology and Technology (SMaRT), KU Leuven, Celestijnenlaan 200J, 3001 Leuven, Belgium

**Keywords:** Ultrasonic microreactors, Emulsification, Cavitation, O/W emulsion, Pits, Ultrasound frequency

## Abstract

An ultrasonic microreactor with rough microchannels is presented in this study for oil-in-water (O/W) emulsion generation. Previous accounts have shown that surface pits or imperfections localize and enhance cavitation activity. In this study cavitation bubbles are localized on the rough microchannels of a borosilicate glass microreactor. The cavitation bubbles in the microchannel are primarily responsible for emulsification in the ultrasonic microreactor. We investigate the emulsification mechanism in the rough microchannels employing high-speed imaging to reveal the different emulsification modes influenced by the size and oscillation intensity of the cavitation bubbles. The effect of emulsification modes on the O/W emulsion droplet size distribution for different surface roughness and frequency is demonstrated. The positive effect of the frequency on minimizing the droplet size utilizing a reactor with large pits is presented. We also demonstrate microreactor systems for a successful generation of miniemulsions with high dispersed phase volume fractions up to 20%. The observed emulsification mechanism in the rough microchannel offers new insights into the utility and scale-up of ultrasonic microreactors for emulsification.

## Highlights

• Emulsification mechanism in a microreactor with rough microchannels is reported.
• Influence of cavitation bubble size on emulsification and droplet breakup is visually confirmed.
• Influence of frequency on the emulsion droplet size is highlighted.
• Optimal setup for miniemulsion generation is demonstrated.

## 1 Introduction

Emulsions are a dispersion of two immiscible liquids where the droplets of one liquid, the dispersed phase, are distributed in another liquid, the continuous phase [1], [2]. Emulsions have various applications in the cosmetic, food, biomedical, and pharmaceutical industry [3], [4], [5], [6]. Emulsion generation typically requires external shear forces to rupture the liquid interface [2]. High energy input is required for the interface breakup to generate emulsion droplets in the submicron range [7], [8], [9], [10]. Amongst other approaches, the application of ultrasound can provide the required large energy input for emulsification. Thus, ultrasound is widely utilized to generate oil-in-water (O/W) or water-in-oil (W/O) emulsions.

In the low-frequency regime (f < 100 kHz) it is the cavitation bubbles generated on sonication that contribute to emulsification. Li and Folger were the first to propose a two-step emulsification mechanism for a batch emulsification setup operated at 20 kHz [11], [12]. The first step involves the disturbance of the oil interface due to the Rayleigh-Taylor instability and the eventual breakup leading to oil droplets in water. The second step is the cavitation bubble collapse in the aqueous phase resulting in further breakup of the oil droplets. Perdih *et al.* proposed an additional step in the emulsification mechanism, which is the formation of a W/O emulsion in the oil phase due to acoustic streaming preceding the Rayleigh-Taylor instability of the oil interface [13]. Further investigations on the role of cavitation bubble collapse in emulsification have shown that the jet-directionality on a bubble collapse is directed from the lighter liquid phase to the denser liquid phase [14], [15], [16], [17]. Furthermore, a bubble collapse in the lighter liquid phase, close to the interface, leads to significant emulsification [14]. Apart from the transient cavitation, the vigorous oscillation of a cavitation bubble in the vicinity of an oil droplet could also lead to droplet breakup [18].

In contrast to the cavitation bubble collapse in the batch emulsification setup, the surface and volume oscillation of a cavitation bubble has been reported to contribute significantly to emulsification in ultrasonic microreactors [19], [20], [21], [22], [23], [24], [25]. Cavitation bubble or bubble cluster oscillation close to the oil–water interface contributes to emulsification in a microchannel [19], [20], [22]. Some reports suggest that the cavitation bubbles shuttle through the oil slug and are encapsulated with an oil layer [23], [25], [26]. The bubble oscillation in the aqueous phase leads to the generation of oil droplets in water. High-frequency ultrasound (f > 1 MHz) has also been successfully applied for emulsification in batch and microreactors [27], [28], [29], [30]. For this frequency range, the droplet breakup results from the surface destabilization of the interface in the absence of any cavitation activity.

It is evident from the previous studies that the stable or transient cavitation activity contribute to emulsification. In addition to this there are also indications that trapping or controlling cavitation bubbles could prove beneficial in harnessing their potential for emulsification. Bai *et al.* investigated the influence of a pit structure (d = 1 mm) on a glass substrate and observed that the pit captured wandering cavitation bubbles and ejected microbubbles [31]. Rivas *et al.* reported an enhancement in the cavitation and sonochemical activity when increasing the number of micromachined pits (d = 30 μm) on a hydrophobic silicon substrate [32], [33], [34], [35]. Rivas *et al.* successfully harnessed the micro-pits in a ‘Cavitation Intensifying Bag’ for emulsification in an ultrasonic bath [36].

The pits on the surface have been shown to localize the cavitation activity and enhancing it. In order to take advantage of the interplay between localized cavitation bubbles and the dispersed oil phase, we investigate novel ultrasonic microreactors with different surface roughness for O/W emulsion generation. Firstly, we report the influence of microchannel surface roughness on the emulsification mechanism modes and the emulsion droplet size for a borosilicate glass ultrasonic microreactor. We also correlate the various emulsification modes observed in the microreactor to the emulsion droplet size for O/W emulsions. Secondly, we investigate the emulsification mechanism and the droplet size in the frequency range of 40–600 kHz. The role of the frequency in the droplet breakup and the eventual influence on the droplet size is reported. Finally, we look at the optimal reactor setup for the generation of miniemulsion (50 nm < d < 1000 nm), which are finding increasing applications in the pharmaceutical and biomedical industry. This mechanistic study will further enhance our understanding of the role cavitation bubbles play in emulsification and provide avenues to optimize the desired emulsion droplet size utilizing microreactors.

## 2 Materials and methods

### 2.1 Materials

The continuous aqueous phase was prepared by mixing Milli-Q water and Tween 20 (Sigma Aldrich) for Tween 20 concentration of 3 wt%. The dispersed phase for the emulsion is decane (Sigma Aldrich), hexadecane (Sigma Aldrich) or store-bought sunflower oil. The physical properties of the liquids are listed in Table 1. Fluorescent polystyrene particles (Spherotech) of 10 μm were used for particle focusing experiments.

**Table 1:** The physical properties of water, decane, hexadecane, and sunflower oil [37], [38], [39], [40].

Fluid	Viscosity @ 30 °C (mPas)	Surface Tension @ 30 °C (mN/m)	Refractive Index
Water	0.82	71.20	1.33
Water + Tween 20	–	37	–
Decane	0.79	23.35	1.41
Hexadecane	2.76	27.05	1.43
Sunflower Oil	42.85	52.70	1.47

### 2.2 Reactor setup

**Fig. 1:**
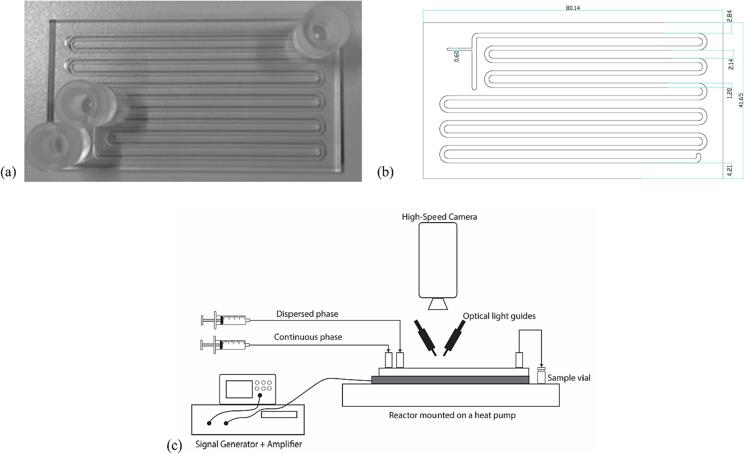
(a) Borosilicate glass reactor with two inlet ports and one outlet port bonded onto the top layer. (b) Sketch of the glass reactor with dimensions in mm. (c) Schematic of the setup to acquire high-speed images and videos for the emulsification mechanism study in the glass microreactors.

Two different reactors were used to investigate the influence of surface roughness on emulsification. Both reactors, fabricated by Little Things Factory GmbH, consist of three borosilicate glass layers. The bottom and the top layer of 1 mm thickness sandwich the middle layer of 1.2 mm thickness, in which a serpentine channel with a cross-section of 1.2 × 1.2 mm^2^ and length of 700 mm is etched. The reactor volume is 1 ml. The channel etching technique influences the final roughness of the channel wall. In the first reactor, the channel was etched by a waterjet cutting technique, resulting in a channel with an average roughness (R_a_) of approximately 4 μm. In the second reactor, the channel was etched by a laser etching technique to reduce the roughness (R_a_) to approximately 1 μm. Two glass inlet ports and an outlet port with a UNF ¼-28″ thread were bonded onto the top layer of the reactor (see Fig. 1(a) and (b)).

The glass microreactor was bonded to a piezoelectric plate transducer (Pz26, Ferroperm) at the bottom using epoxy glue (EpoTek 301). The reactor was coupled with a piezoelectric plate transducer of thickness 1.67 mm or 4 mm (80 × 40 × 1.67 mm^3^ & 80 × 40 × 4 mm^3^) to actuate the reactor at one of the resonance frequencies (Fig. S1). The admittance was measured using an Impedance Analyser (16777 k, SinePhase) to obtain the resonance frequencies and, therefore, the operating frequencies. The reactor coupled to the piezo plate with a thickness of 1.67 mm was operated at the resonance frequencies of 48 kHz or 142 kHz (Fig. S1(a)). The reactor coupled to the thicker piezo plate of 4 mm was operated at the resonance frequencies of 310 kHz or 525 kHz (Fig. S1(b)).

Ultrasound was actuated by connecting the reactor to a signal generator (33500B, Keysight) connected to a power amplifier (RF 1040L, 400 W, E&I), which generated a sinusoidal wave of desired frequency and input load power. The reactor was placed on a Peltier Cooling Element (RS Components) connected to a DC power supply (Velleman) to regulate the reactor outlet temperature during the experiments. The aqueous and the oil phase were supplied to the reactor using two syringe pumps (Fusion 200, KR Analytical), with PFA tubing (OD 1/16″, ID 1 mm, IDEX) aiding the transfer of the two phases to the reactor and from the outlet.

### 2.3 High speed imaging

The emulsification mechanism in the glass microreactor was observed with an optical microscope (SMZ25, Nikon) connected to a high-speed camera (Mini UX100, Photron). The reactor channel was illuminated from the top with a two-branch optical light guide (KL Series, Schott AG) connected to a LED light source (KL 2500 D, Schott AG). The schematic of the setup for video acquisition is shown in Fig. 1(c). The high-speed videos and images were processed later using Photron FASTCAM Viewer PFV4 (ver 4.0.3.2) and ImageJ [41].

### 2.4 Droplet size distribution

The continuous phase flow rate and the dispersed phase flow rate for the emulsion generation was 0.2 ml/min and 0.05 ml/min, amounting to a total flow rate of 0.25 ml/min and a residence time of 4 min unless otherwise stated. An ultrasonic load power of 20 W (energy density: 4.8 × 10^8^ J/m^3^) was applied for emulsion generation and the temperature at the outlet was maintained at 30 °C. The droplet size of the O/W emulsion was measured using laser diffraction (Mastersizer 3000, Malvern). The O/W emulsion sample was collected in an HPLC vial after 3 reactor residence times. Approximately 5–20 μl O/W emulsion was introduced immediately after collection into the measurement cell filled with 7 ml of Milli-Q water. The sample was stirred at 1000 rpm during the measurement. The droplet size measurement was repeated thrice for every condition, and an average droplet size distribution is reported.

## 3 Results and discussion

### 3.1 Influence of surface roughness

**Fig. 2:**
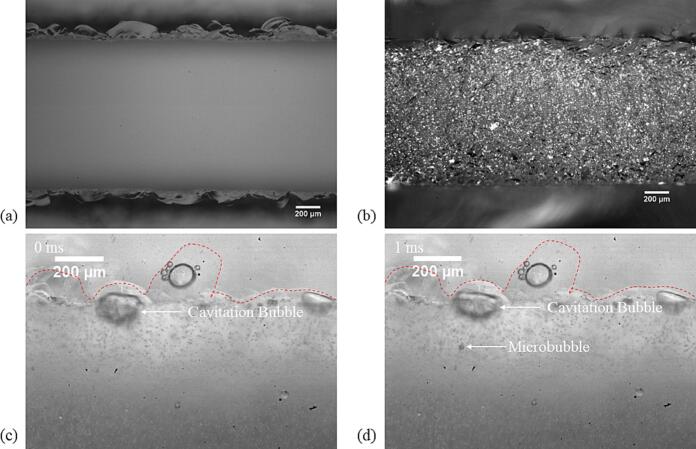
(a) Top view of the waterjet cut microreactor, with large pits visible at the top end of the side wall. (b) Side view of the waterjet cut microreactor, with small pits visible in the middle region of the side wall (Ra ∼ 4 μm). (c) Cavitation bubble situated in a pit on the channel side wall. The ultrasound frequency is 48 kHz, and the load power is 10 W. (d) Microbubble ejection from the cavitation bubble at t = 1 ms. The outline of the microchannel is shown with a dashed line in (c) and (d).

The waterjet cut microreactor (WJR) channel side walls have a significant number of small pits in the middle region of the side wall, and large pits measuring a few hundred micrometres in length and depth at the top end of the side walls, as seen in Fig. 2(a) and (b). The pits or the imperfections on the channel wall can act as initial nucleation site for the cavitation bubbles [42]. On ultrasound actuation at 48 kHz, the gas nuclei undergo compression and expansion and grow due to rectified diffusion [42]. The cavitation bubbles generated on the rough microchannel also migrate along the channel wall and settle in other pits. This is in line with the previous observation on the ability of a channel imperfection to trap a cavitation bubble in a microreactor [31]. The cavitation bubbles also eject tiny gas bubbles (see Fig. 2(c) and (d)) when growing to a radius closer to or larger than the theoretical linear resonance radius at 48 kHz (R_r_ ∼ 56 μm) given by Eq. (1) [31], [43].

(1) $$R_{r}=\;\frac{1}{2\pi f}\sqrt{\frac{2\gamma P_{h}}{\rho }}\;$$

**Fig. 3:**
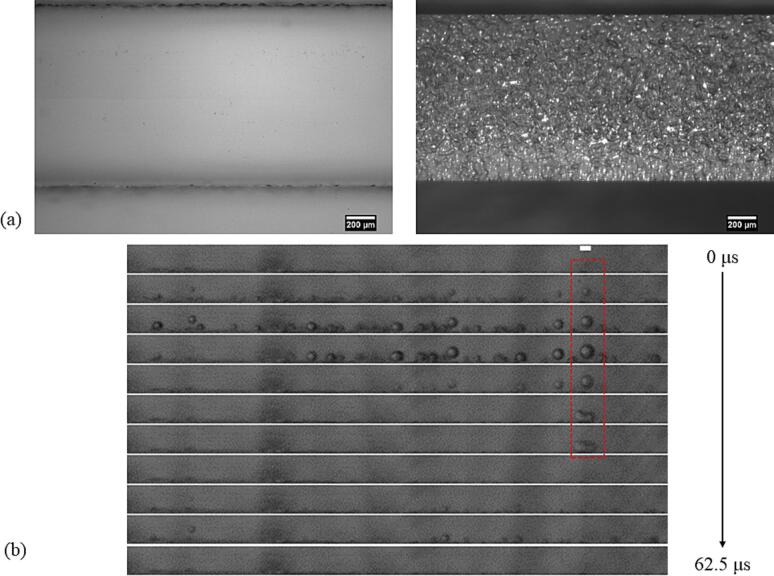
(a) Channel side wall of the laser etched microreactor (LCR). The top view (left) shows pits with depths of 10–20 μm and the side view (right) shows pits with length and breadth of 30–60 μm. (b) Extracted frames from the high-speed video of the transient cavitation on the channel wall at 48 kHz. The time step between each frame is 6.25 μs. Scale bar = 100 μm. The image is enhanced by changing the brightness to better visualize the cavitation bubbles.

Here, *γ = C_p_/C_v_* denotes the ratio of the specific heat of the gas at a constant pressure to its specific heat at a constant volume, *P_h_* the hydrostatic liquid pressure, *ρ* the liquid density, and *f* the ultrasound frequency. Bai *et al.* also reported a similar phenomenon where the cavitation bubbles trapped in concave pits eject tiny gas bubbles, also referred to as microbubbles [31]. The cavitation bubble in a concave pit ejects a microbubble during collapse or bubble contraction. Zijlstra *et al.* also reported ejection of pinch-off bubbles from a cavitation bubble situated in a cylindrical pit. They proposed that the bubble pinch-off occurred due to ‘folding’ of the gas liquid interface resulting from the bubble oscillation with a large amplitude capillary wave. As can be seen in Fig. 3(b) and (c), the cavitation bubble undergoes chaotic surface oscillation, which would result in break-up of gas nuclei from the bubble. The ejected gas bubbles (microbubbles) move rapidly in the channel, eventually settle in a pit, and continue to grow.

The laser etched microreactor (LCR) has pits on the channel walls, which are shallower and smaller than the pits in the WJR (See Fig. 3(a)). The pit depth ranges from 10 to 20 μm while the length or width is 30–60 μm. On ultrasound actuation, transient cavitation bubbles are observed in the channel as seen in Vid 1. The transient cavitation bubble could result from gas nuclei or small gas pockets in the channel pits, which expand till reaching the critical resonance radius and collapse [44]. Fig. 3(b) depicts extracted frames from the high-speed video (160,000 fps) with a time step between each frame of 6.25 μs, which is one-third the time for a full acoustic cycle at 48 kHz. The gas nuclei in frames 2–6, close to the pits on the channel wall, expand and contract. As seen in Vid. 1, the gas bubble clusters undergo a continuous cycle of expansion and contraction close to the channel wall. Previous studies on the role of pits of with precise dimensions and depths of 10–30 μm on a silicon substrate show an increase in transient cavitation activity in the pit as well as in its close vicinity compared to the areas without pits [32], [33], [34]. This cavitation activity in the presence of the pit was referred to as ‘bubble cloud’ [32]. Moreover, increasing the number of pits led to an increase in transient cavitation activity and interaction between the cavitation bubble clouds [32], [33], [34]. In addition, the micro-jet on multibubble collapse is directed parallel to the surface [45]. The cavitation activity observed in the LCR could well be in-line with the cavitation bubble clouds observed for silicon substrates with pits. The cavitation bubble clusters could undergo a collapse with the micro-jet direction parallel to the rough microchannel. A single bubble is also seen to collapse asymmetrically leading to a micro-jet directed towards the channel wall (red box in Fig. 3(b) and Vid. 1). A single gas bubble in the vicinity of a rigid wall undergoes asymmetric collapse with the micro-jet directed towards the wall [46]. This could explain the micro-jet directed towards the channel wall observed for some cavitation bubbles in the LCR. Along with the transient cavitation bubble cloud, cavitation bubbles undergoing chaotic oscillation and ejecting microbubbles are also observed on the microreactor channel wall.

Earlier studies suggest that stable cavitation bubbles, generated by ultrasound, migrate freely through the microchannel and are majorly situated in the microchannel [19], [20], [23], [24]. Contrary to the previously studied reactor setups with smooth microchannels, the cavitation bubbles occupy the pits on the channel wall in both the WJR and the LCR. As expected, the pits are effective in trapping and harnessing the cavitation activity in certain regions in the microchannel. In the WJR, we typically see cavitation bubbles which grow from small gas nuclei in the pits or from ejected microbubbles. These cavitation bubbles typically undergo periodic or irregular shape oscillation in the microchannel. This cavitation activity, wherein the cavitation bubbles undergo volume or shape oscillation in the microchannel, is termed ‘stable cavitation’. In the LCR, we observe that the gas nuclei undergo rapid expansion and contraction close to the channel wall. This cavitation activity, wherein the bubbles have a short lifetime and eventually collapse, is termed ‘transient cavitation’. A ‘stable cavitation’ activity is primarily observed in the WJR, while ‘transient cavitation’ activity dominates in the LCR. Thus, either stable or transient cavitation activity dominates, depending on the channel pit size. The emulsification mechanism is studied in the WJR and the LCR to understand the role of stable and transient cavitation bubbles in the generation of emulsion droplets and their influence on the emulsion droplet size.

The interaction of the cavitation bubbles localized on the channel wall with the segmented flow of sunflower oil–water and their role in emulsification is studied in the entrance region of the microchannel. High-speed images are recorded right after the actuation of ultrasound at 48 kHz and 10 W since the emulsion quickly turns opaque, making it difficult to observe the emulsification mechanism.

**Fig. 4:**
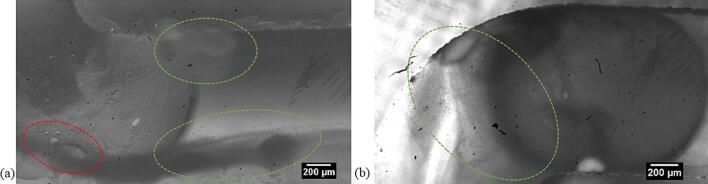
Emulsification of the sunflower oil slug at the entrance of the (a) WJR and (b) LCR at 48 kHz. The slug is emulsified to minuscle oil droplets (encircled in green), also defined as fine dispersion, and large droplets (encircled in red).

The stable or transient cavitation bubbles on the channel wall undergo stable or chaotic oscillation and, on interaction with an oil slug, generate oil droplets in water. Depending on the position of the cavitation bubble relative to the oil–water interface and the oscillation mode, droplets of various sizes are generated in the microchannel (see Fig. 4). We can classify them into fine dispersion, i.e., minuscule droplets dispersed in the aqueous phase (droplets in the green enclosure) and large droplets (droplets in the red enclosure). As the sunflower oil slug moves along the channel, the cavitation bubbles break it down into fine dispersion or large droplets. The mechanism of the fine dispersion or large droplet generation is further highlighted by closer investigation of the cavitation bubbles and the dispersed phase interplay.

**Fig. 5:**
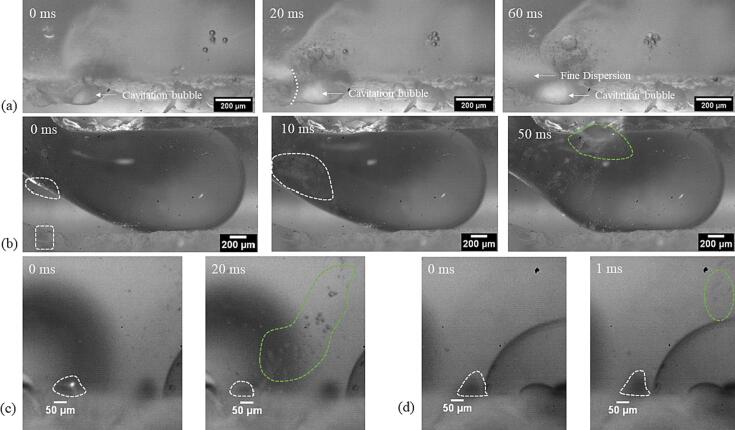
Emulsification modes in the WJR. The timestamp in milliseconds (ms) is indicated in the top left corner (a) Fine dispersion generation on the channel wall. The cavitation bubble on the channel wall oscillates and is encapsulated by an oil layer at 20 ms. The violent oscillation generates a fine dispersion. (b) A cavitation bubble situated in a pit (white dashed rectangle) ejects microbubbles which enter the oil slug (white dashed region on the oil slug). The microbubbles cluster (white dashed region at 10 ms) and oscillate violently to generate a fine dispersion (green dashed region at 50 ms). (c) An oil-encapsulated cavitation bubble (white dashed line at 0 ms) undergoes weak oscillation to generate large droplets (droplets inside green dashed line at 20 ms). (d) An oil-encapsulated cavitation bubble (white dashed line at 0 ms) ejects large oil droplets into the aqueous phase (droplets inside green dashed line at 1 ms).

A major fraction of the cavitation bubbles situated in the pits in the WJR oscillates violently. When these bubbles come close to the oil–water interface, they are encapsulated by an oil layer. One such example is shown in Fig. 5(a) and Vid 2. The cavitation bubble in the pit (Fig. 5(a) 0 ms) is encapsulated by a layer of oil (Fig. 5(a) 20 ms) as the oil slug moves along the channel. The violent oscillation leads to the generation of a fine dispersion, as seen in Fig. 5(a) at 60 ms. In many instances, the cavitation bubbles also shear off a droplet from the oil slug in motion, and the droplet is broken down into finer droplets due to the chaotic oscillation of the cavitation bubbles.

It was also observed that the microbubbles ejected from the cavitation bubbles in the pits move across the channel through the oil slug, coalesce or cluster, and oscillate violently to generate fine dispersion, as seen in Fig. 5(b), Vid 3, and Vid 4. On actuation of ultrasound, the tiny gas pockets in the pits on the channel wall grow due to rectified diffusion [44], [47], [48], [49]. A cavitation bubble, seen in Vid 3, starts ejecting microbubbles and the microbubbles migrate to the oil interface (Fig. 5(b) 0 ms). The microbubbles cluster together in the oil slug or on the oil interface (Fig. 5(b) 10 ms). The microbubble cluster eventually moves to the opposite wall and their chaotic oscillation leads to the generation of a fine dispersion (Fig. 5(b) 50 ms). Similarly, in the LCR, cavitation bubble clusters move rapidly through the oil slug to the channel wall and the chaotic oscillation leads to the generation of a fine dispersion (see Vid 4).

Apart from the violent oscillation, the cavitation bubbles also undergo weak oscillation when their radius is smaller than the linear resonance radius [47], [50], [51], [52]. On the weak oscillation of the oil-encapsulated cavitation bubbles, large droplets (typically d > 5 μm) break off from the oil layer, as can be seen in Fig. 5(c) and Vid 5. At 0 ms, an oil-encapsulated cavitation bubble is observed on the channel wall (denoted by the white dashed line). As it oscillates, oil droplets shear off from the oil interface and are ejected into the continuous aqueous phase (droplets in the green dotted line), as seen at 20 ms in Fig. 5(c). These observations are in agreement with previous accounts of emulsification via weak oscillation of an oil-encapsulated bubble [23], [26].

For some oil-encapsulated bubbles in the WJR, oil droplets were seen to be ejected at high velocity as seen in Vid 6. A previous report suggests that a cavitation bubble in a well-defined pit ejects bubbles with a diameter of 2–10 μm at a typical velocity of 2 m/s at the pinch-off event [34]. A similar phenomenon could explain the ejection of oil droplets from the oil-encapsulated bubble trapped in a pit. An oil-encapsulated bubble at 0 ms ejects a few droplets similar at high velocity as seen in the Vid 6 and Fig. 5(d). The droplets travel approximately 400 μm in 1 ms, which corresponds to an average velocity of 0.4 m/s. The droplet breakup event that is observed for some oil-covered bubbles could lead to the ejection of emulsion droplets of a few micrometers in diameter.

The transient cavitation bubble clouds in the LCR also contribute to the generation of large droplets, as seen in Vid 7. The transient cavitation bubble cloud close to the oil–water interface migrates away from the oil slug. An oil filament moves along with the bubble cloud and breaks into large droplets. The oil droplet eventually resettles on the channel wall and undergoes further breakup.

A mechanistic study was also carried out for the decane-water system in the WJR and the LCR. A similar mechanism is observed in the WJR, albeit, with more occurrences of fine dispersion generation. In the LCR, the fine dispersion generation is accompanied by the generation of large droplets due to transient cavitation bubble clouds close to the interface, as seen in Vid 8. The micro-jets on the cavitation bubble collapse in the bubble cloud are directed either parallel to the channel wall or towards the decane-water interface. The decane interface undergoes severe deformation and breaks into large droplets along with a fine dispersion.

The strong cavitation microstreaming in a microchannel would also contribute to the breakup of the large emulsion droplets into smaller ones [18], [24]. In the WJR, it is observed that droplets larger than the cavitation bubble undergo breakup while this is not the case for the ones smaller than the cavitation bubble. In case of a droplet larger than the cavitation bubble, it moves closer to the oscillating bubble, encapsulates the bubble and ruptures into smaller droplets. In case the droplet is smaller than the oscillating cavitation bubble, the droplet moves rapidly due to the cavitation microstreaming when closer to the bubble. The droplet eventually escapes the microstreaming without any visible breakup or size change. A similar observation was made for the droplet breakup in the LCR. The droplet breakup in the vicinity of transient cavitation bubbles is studied in detail and is illustrated below.

**Fig. 6:**
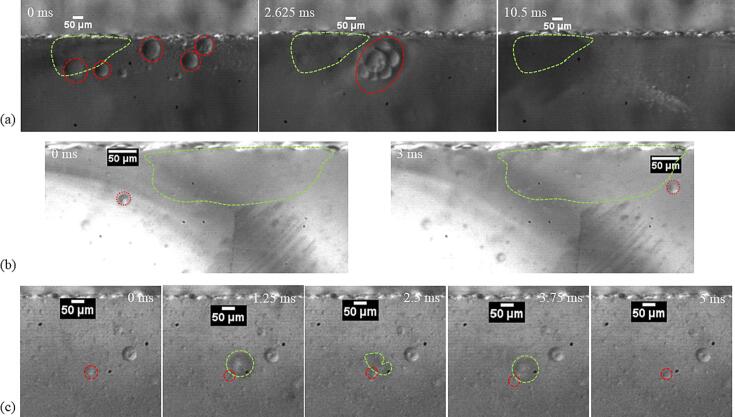
(a) The large decane droplets (D > 60 μm) close to the microstreaming of the transient bubble cloud (in the green dashed region), bunch together and undergo breakup into smaller droplets. The timestamp in milliseconds (ms) is indicated in the top left corner. (b) The sunflower oil droplet (D = 21 μm, encircled in red dashed circle) close to the transient cavitation bubble cloud (inside green dashed line) moves rapidly through the bubble cloud and retains its size. The timestamp in milliseconds (ms) is indicated in the top left corner. (c) The decane droplet (D = 32 μm, encircled in red dashed line) is in the close vicinity of a collapsing cavitation. The droplet does not undergo any visible breakup. The timestamp in milliseconds (ms) is indicated in the top right corner.

Decane droplets in the size range of 60 to 90 μm, as seen in Fig. 6(a) and Vid 9 rupture into smaller droplets in the vicinity of the transient cavitation bubbles. The radius of the droplets in this case is larger than the linear resonance radius of the cavitation bubbles at 48 kHz. The oil and the decane droplets smaller than the cavitation bubbles did not undergo any visible breakup in the close vicinity of the collapsing bubble. In Vid 10, the oil droplet measuring 21 μm enters the transient cavitation bubble cluster. The droplet retains its size even after experiencing high shear stress in the close vicinity of the transient cavitation bubbles, as seen in Fig. 6(b). Similarly, a decane droplet of a diameter of approximately 32 μm next to a collapsing bubble does not decrease in size (see Fig. 6(c)). Other decane droplets close to the transient cavitation bubbles also do not undergo further breakup when they are smaller than the cavitation bubble, as seen in Vid 11.

Recent simulations and experiments have shown that on bubble collapse the jet is directed in the direction of the denser phase [14], [15], [17]. These studies focused on the interaction of a single cavitation bubble with an oil droplet where the droplet diameter was an order of magnitude larger than the cavitation bubble diameter. Significant emulsification was only observed in the case of a cavitation bubble collapse in the lighter phase [14], [15]. These observations are also valid for no breakup for the droplets smaller than the cavitation bubbles in the event of cavitation bubble collapse in the vicinity of the droplet. In case of a droplet larger than a cavitation bubble, the acoustic streaming could lead to the formation of W/O emulsion in the oil droplet [14]. The water droplet could contain some gas nuclei, which on expansion and collapse could lead to the droplet breakup and emulsification. In addition, the high shear stress experienced by the large droplets in the vicinity of a strongly oscillating cavitation bubble could lead to further breakup [18]. The high-speed images reveal that the dispersed phase droplets with sizes larger than the cavitation bubble in the microchannel undergo further breakup while the ones smaller than the cavitation bubble experience minimal or no breakup.

The generation of a wide range of droplets, as identified in this study, could have a major influence on the final droplet size distribution. The influence of these mechanistic observations on the droplet size is quantified for the decane-in-water (D-W), hexadecane-in-water (H-W), and sunflower oil-in-water (O-W) emulsions. The O/W emulsions are generated at a frequency of 48 kHz and a load power of 20 W in the WJR and the LCR. The continuous and the dispersed phase flow rate is 0.2 ml/min and 0.05 ml/min respectively, for a residence time of 4 min in the reactor.

**Fig. 7:**
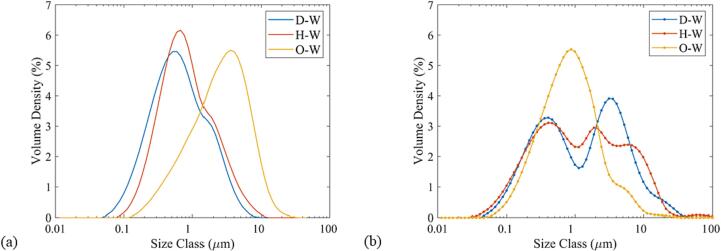
Droplet size distribution of the sunflower oil-in-water (O-W), hexadecane-in-water (H-W), and decane-in-water (D-W) emulsions in (a) waterjet cut microreactor (WJR) and (b) laser etched microreactor (LCR). The emulsion is generated at a load power of 20 W and frequency of 48 kHz. The continuous phase (water + Tween 20(3 wt%)) flow rate is 0.2 ml/min, and the dispersed phase flow rate is 0.05 ml/min. The residence time is 4 min.

The droplet size distributions of the D-W and H-W emulsions are unimodal for the WJR while bimodal for the LCR, as seen in Fig. 7. A peak below 2 μm represents the fine dispersion of decane and hexadecane in the aqueous phase. The other peaks in the LCR and the slight bump close to 2 μm in the WJR for D-W and H-W correspond to droplets between 1 and 30 μm generated due to weakly oscillating cavitation bubbles or transient cavitation. These droplets are smaller than the linear resonance bubble radius of 56 μm at 48 kHz and do not undergo further breakup. The droplet size distribution for D-W and H-W, therefore, confirms the previous mechanistic observations of the generation of a fine dispersion and large droplets, as well as the influence of the cavitation bubble size on the droplet breakup.

The O-W emulsion generated employing the LCR has smaller droplets compared to the ones generated using the WJR at 48 kHz. The droplet size distribution indicates that the fine dispersion makes up the major fraction in the LCR, while the large droplets are the major fraction in the WJR. The droplet size distribution for O-W is also in-line with the observations of the influence of cavitation bubble size on the droplet breakup, with the major fraction of the droplets below 56 μm.

The droplet size distribution and the D_10_, D_50_, and D_90_ (see Fig. S2) also reveal that for dispersed phase with a high viscosity, like sunflower oil, transient cavitation and cavitation bubble clouds can help in minimizing the emulsion droplet size. A lower viscosity of the dispersed phase, as for decane and hexadecane, favours stable cavitation for the generation of smaller emulsion droplets.

**Fig. 8:**
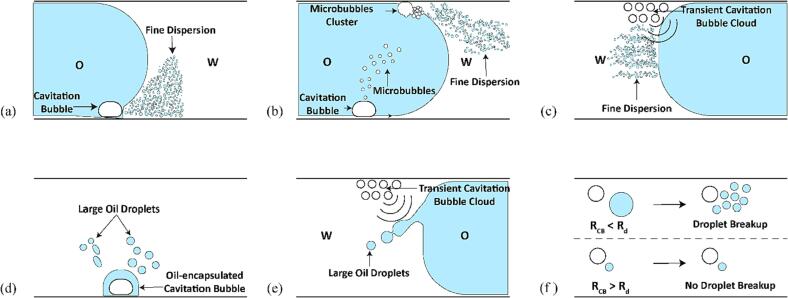
Schematic diagram of the different emulsification modes observed in the WJR and LCR. (a) Generation of a fine dispersion due to chaotic oscillation of a cavitation bubble on the channel wall in the WJR. (b) Generation of a fine dispersion due to a microbubble cluster situated on the channel wall in the WJR and the LCR. (c) Generation of a fine dispersion due to a transient cavitation bubble cloud close to the oil interface in the LCR. (d) Generation of large droplets due to the oscillation of an oil-encapsulated cavitation bubble. (e) Generation of large droplets due to a transient cavitation bubble cloud situated on the channel wall in the LCR. (f) Droplet breakup in a microreactor when the cavitation bubble is smaller than the oil droplet (R_CB_ < R_d_), and no droplet breakup when the cavitation bubble is larger than the oil droplet (R_CB_ > R_d_).

To summarize, the mechanistic study points to two modes of emulsification in a microreactor. The different emulsification modes are summarized in Fig. 8. The first mode, generation of a fine dispersion, results from the chaotic cavitation bubble or bubble cluster oscillation on the channel wall in the WJR and the LCR (Fig. 8(a) and (b)). In addition, a fine dispersion generation is also observed due to transient cavitation in the LCR (Fig. 8(c)). The second mode, generation of large droplets, result from the weakly oscillating cavitation bubbles in the WJR (Fig. 8(d)). In the LCR, the transient cavitation bubble cloud located close to the interface also contributed to generation of large droplets (Fig. 8(e)) due to strong micro-jets generated on bubble collapse. In addition, only the droplet size larger than the cavitation bubbles were seen to undergo further breakup in the WJR and the LCR (Fig. 8(f)). Overall, the mechanistic study clearly reveals that the cavitation bubble oscillation amplitude, size, and its position play an important role in determining the size of the oil droplets generated in the microchannel.

### 3.2 Influence of frequency

The rough microchannel acts as the preferential sites for the cavitation activity and emulsification in the glass microreactor. Here, we investigated the WJR, which exhibits primarily stable cavitation bubbles. The emulsification was studied at frequencies higher than 100 kHz, that resulted in smaller cavitation bubbles.

**Fig. 9:**
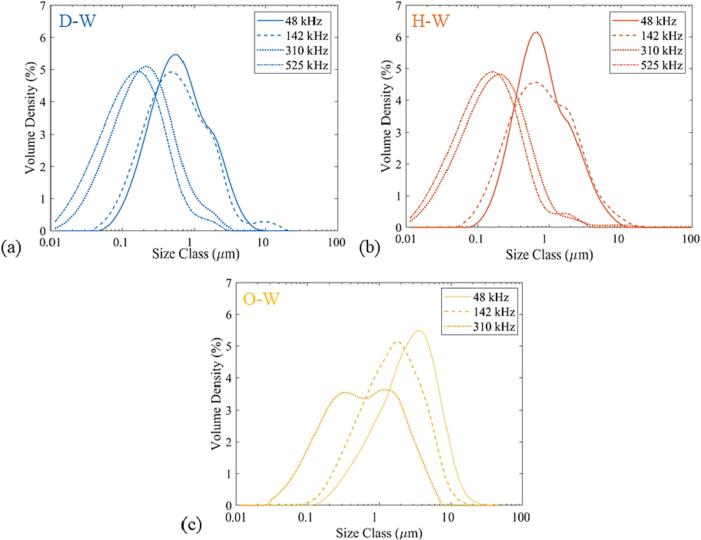
Droplet size distribution for the decane-in-water (a), hexadecane-in-water (b), and sunflower oil-in-water (c) emulsion at frequencies of 48 kHz, 142 kHz, 310 kHz, and 525 kHz. The aqueous phase flow rate is 0.2 ml/min, the dispersed phase flow rate is 0.05 ml/min, and the applied load power is 20 W. The sunflower oil did not undergo emulsification at 525 kHz and thus the droplet size distribution is not shown in the figure.

The WJR, coupled to a piezoelectric plate transducer of thickness 1.67 mm or 4 mm, exhibits additional resonance frequencies at 142 kHz, 310 kHz and 525 kHz respectively (Fig. S1). The emulsification mechanism at 142 kHz and 310 kHz for the decane-water and sunflower oil–water systems was similar to the one observed at 48 kHz. The droplet size distribution for D-W, H-W, and O-W emulsions resulting from the emulsification mode of fine dispersion and large droplets at 20 W is shown in Fig. 9. The difference in the droplet size distribution between 48 kHz and 142 kHz is negligible for D-W and H-W (See Fig. 9 and Fig. S3). The O-W emulsification at 142 kHz results in smaller droplets compared to 48 kHz. It is important to note that for all the dispersed phases, the majority of the emulsion droplets are smaller than the linear resonance bubble radius (R_r_ ∼ 19 μm) at 142 kHz. Interestingly, the trend of the droplets being smaller than the linear resonance bubble radius (R_r_ ∼ 8.7 μm) is also observed for the frequency of 310 kHz.

It is apparent from the droplet size distribution that the droplets generated by the fine dispersion mode (d < 1 μm) decrease in size and increase in volume fraction as the frequency increases. The high-speed imaging could not offer an explanation for this decrease in the droplet size. A possible explanation might be the increase in number and oscillation intensity of the strongly oscillating cavitation bubbles at a higher frequency. Sonoluminescence studies have shown an increase in the number of cavitation bubbles at higher ultrasound frequencies in the range 20–600 kHz [53], [54]. In addition, the increase in frequency leads to an increase in the acoustic streaming intensity for a cavitation bubble of the same radius as it approaches the resonance radius [55]. Since the size of the cavitation bubbles is primarily in close proximity to the linear resonance radius in a microchannel, the increase in frequency could lead to an increase in the cavitation bubble number and the oscillation strength [51]. This eventually could result in a decrease in the droplet size and increase in the volume fraction of the fine dispersion at higher operating frequency. In addition, as stated in the previous section, the droplets larger than the cavitation bubbles will undergo breakup in the microchannel. Thus, smaller cavitation bubbles resulting at higher frequency would also lead to a decrease in the overall droplet size of the final emulsion due to breakup of the larger droplets. This is also evident from the decrease in the D_90_ (Fig. S3) and droplet size distribution for the O/W emulsions at higher frequency.

**Fig. 10:**
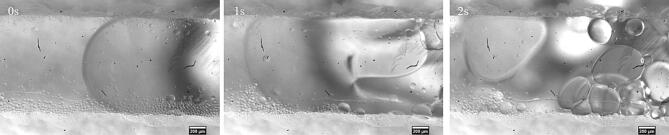
The breakup of a decane slug in the microchannel at 525 kHz and a load power of 10 W. Upon actuation the decane slug undergoes deformation (1 s) and eventually breaks into large droplets of few hundred micrometres (2 s).

**Fig. 11:**
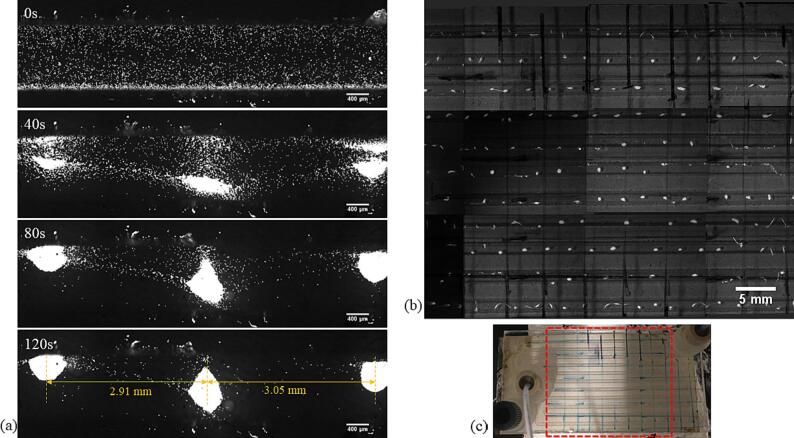
(a) The fluorescent polystyrene particles spread evenly at the bottom of the channel. On the actuation of ultrasound at 525 kHz and 1 W, the particles start migrating towards the pressure nodes (40 s) and eventually concentrate at the pressure nodes in the microchannel (80 s). The approximate interval between the two pressure nodes (120 s) is roughly equal to the ultrasound wavelength in the microchannel. (b) The overview of the microreactor with the bright spots corresponding to the particle concentration at the pressure nodes in the microchannel. The area in red in (c) is the pictured microreactor region in (b).

At the higher frequency of 525 kHz, no significant emulsification was observed for the sunflower oil–water system. For the decane-water system, we observed a breakup of the slug in the reactor channel as seen in Fig. 10 and Vid 12. Previous accounts of emulsification at a frequency above 1 MHz point to the role of a standing wave in the microchannel in the breakup of dispersed phase droplets [29], [30]. To determine the presence of a standing wave in the microchannel, we introduced 10 μm fluorescent polystyrene particles in the channel. In the presence of a standing wave, particles or droplets migrate to a pressure node or antinode depending on the contrast factor [56], [57]. Once the particles are introduced in the channel, the flow is stopped, and the particles are allowed to settle down at the bottom of the channel. The ultrasound is activated at 525 kHz and a load power of 1 W to avoid any cavitation in the channel. The particles slowly start moving to certain areas in the channel which correspond to the pressure node in the microchannel (see Vid 13 and Fig. 11(a)). The interval between the pressure nodes corresponds roughly to the ultrasound wavelength (λ_L_) in water (λ_L_ = 2.86 mm). An overview of the serpentine channel, as seen in Fig. 11(b), shows the presence of pressure nodes along the microchannel.

**Fig. 12:**
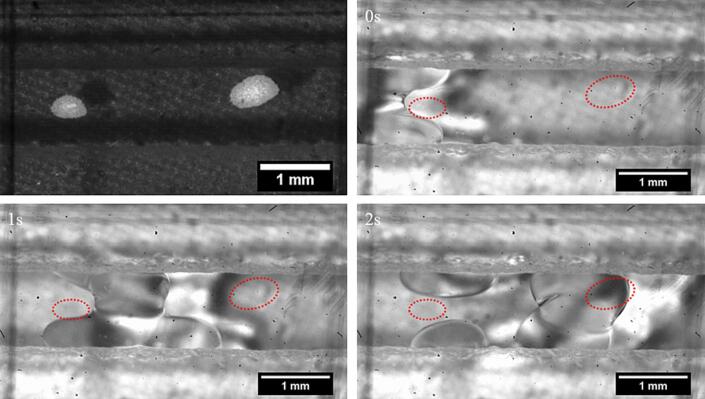
The breakup of a decane slug in a microchannel section with 2 pressure nodes. The top left picture depicts the position of the pressure nodes in the channel. The position of the pressure nodes (and the particle position) is depicted by the dashed red circle in the channel. The slug is deformed as it approaches the first pressure node (0 s) and moves around the pressure node. As it is located between the two pressure nodes (1 s), it undergoes deformation and oscillation. The slug eventually breaks into large droplets (2 s), which occupy the channel wall.

Next, the breakup of the decane slug was observed in the vicinity of the pressure nodes. The oil droplets tend to migrate towards the pressure antinodes in the presence of a standing wave [56]. As a decane slug enters the region in the microchannel with 2 pressure nodes (Fig. 12), the slug undergoes deformation, moves away from the pressure node, and is pinned on the channel walls. Eventually, the slug undergoes severe oscillation and deformation at the possible pressure antinode located between the two pressure nodes in the channel (see Fig. 12 and Vid 14). Two large droplets break off from the slug and settle on the channel wall. Thus, it is apparent that the pressure antinodes in the microchannel are the sites for slug oscillations and breakup. Slug deformation and breakup are also observed in case of hexadecane while no breakup is seen for sunflower oil.

**Fig. 13:**
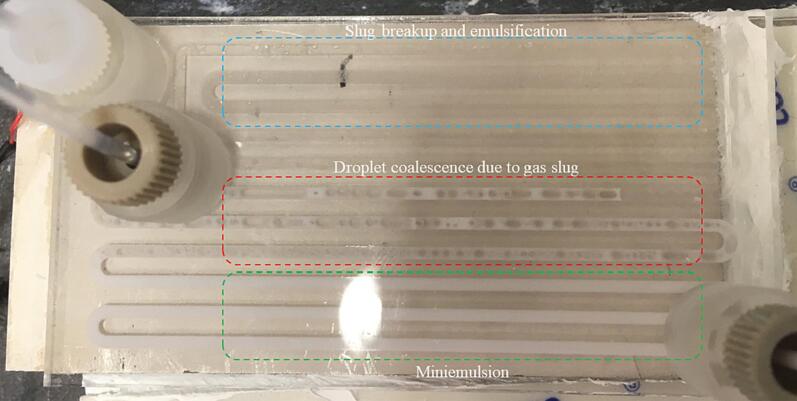
Decane emulsification in the aqueous phase at 525 kHz. In the first section of the reactor (region in blue), the decane slugs undergo breakup and are trapped in the channel. The gas bubbles in the reactor coalesce in the channel to form a gas slug. The slug pushes the droplets not emulsified (region in red) as well as the miniemulsion (region in green and white emulsion in red region).

**Fig. 14:**
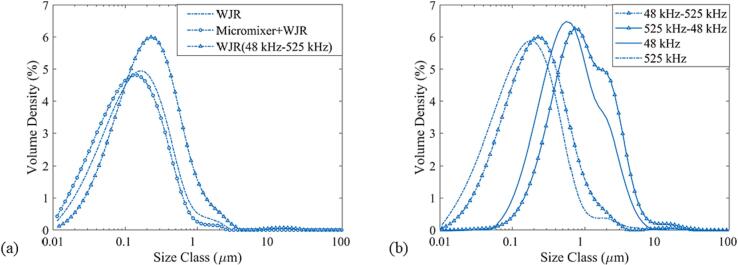
(a) D-W miniemulsion droplet size distribution for the WJR (decane vol% 7.5 %), Micromixer and WJR in series (decane vol% 17 %), and two WJR in series (decane vol% 20 %). The total flow rate is 0.25 ml/min, and the power is 20 W. (b) D-W miniemulsion droplet size distribution for two WJR in series in 2 different configurations in comparison with the WJR operated at a single frequency. The first is 48 kHz WJR followed by 525 kHz WJR and the second is 525 kHz WJR followed by 48 kHz WJR. The single WJR is operated at a total flow rate of 0.25 ml/min, a power of 20 W and a decane volume fraction of 20 %. The WJR in series are operated at a total flow rate of 0.5 ml/min, a power of 10 W for each reactor and a decane volume fraction of 20 %.

The emulsification was incomplete in the microreactor for the continuous and dispersed phase flow rate of 0.2 ml/min and 0.05 ml/min respectively, for a residence time of 4 min, and the load power of 20 W. A closer look at the microchannel could explain the possible reason for incomplete emulsification in the channel. As the slug enters the microchannel, it deforms and ruptures into droplets which are stationary on the channel wall or in the channel. Further, they undergo emulsification due to cavitation bubbles on the channel wall. A small fraction of the cavitation bubbles migrates to the antinodes in the channel and coalesce to eventually form a gas slug. The gas slug, as it moves along the channel, carries the droplets before they are fully emulsified, leading to two sections in the microchannel. The first section is the miniemulsion (d < 1 μm) (channel in green section in Fig. 13) and the second is the section with decane droplets and miniemulsion preceded by a gas slug (channel in red section in Fig. 13). Lowering the volume fraction of the dispersed phase to 7.5 % by changing the continuous and dispersed phase flow rate to 0.23125 ml/min and 0.01875 ml/min for the total flow rate of 0.25 ml/min achieves complete emulsification. The miniemulsion droplet size distribution measured for the volume fraction of 7.5 % and 20 % at 525 kHz did not have any significant differences (See Fig. 14 and Fig. S6). The D-W emulsion was also generated for the volume fraction of 7.5 % at 48 kHz and 20 W to determine if the miniemulsion generation was the effect of the lower dispersed phase volume fraction or of the frequency. From the droplet size distribution (see Fig. S4), it is evident that emulsion droplets at 48 kHz are significantly larger than at 525 kHz. Thus, we can rule out the influence of the lower volume fraction. These droplet size distribution measurements align with the previous observation of decrease in the droplet size at higher frequency.

The investigation of emulsification mechanisms and droplet size for different frequencies reveals that the higher operating frequency in the WJR is beneficial in obtaining smaller emulsion droplets. This is contrary to previous reports for batch and microreactors [20], [22]. In the WJR, the presence of pits promotes cavitation activity in the microreactor. The cavitation bubble size, which decreases with an increase in frequency, also plays an important role in the breakup of droplets in the microreactor. A synergy of these phenomena could be instrumental in minimizing the droplet size in the microreactor with rough channels.

The frequency of 525 kHz led to the smallest emulsion droplets for D-W and H-W and achieved droplet sizes in the miniemulsion range. However, full emulsification could only be achieved at the lower volume fraction of 7.5 %. The next section elaborates on possible approaches for miniemulsion generation at a higher volume fraction of the dispersed phase.

### 3.3 Miniemulsion generation

An emulsion with low dispersed phase volume fraction might suffice for some applications, however, applications in miniemulsion polymerization for nanoparticle synthesis aim for higher dispersed phase volume fractions. Previous studies aiming for miniemulsion generation employing ultrasound are restricted to a volume fraction of 1–15 % [7], [24], [29], [36], [58]. Keeping this in mind, the miniemulsion generation is attempted for a higher volume fraction by coupling the WJR with a micromixer or another WJR in series. The rationale behind the extra emulsification step is to aid the droplet breakup and emulsification in the WJR at 525 kHz before it is influenced by a gas slug.

The first approach was to couple a micromixer (Microreactor Design 3227, Chemxtrix BV) with the WJR. The SOR-mixers in the microchannel generate decane droplets of 50–200 μm (see Fig. S5). The emulsion enters the WJR which is operated at a frequency of 525 kHz and a power of 20 W for the total flow rate and residence time of 0.25 ml/min and 4 min respectively. The micromixer-WJR system was successful in generating a D-W miniemulsion with a dispersed phase volume fraction up to 17 % and a similar droplet size distribution (see Fig. 13(a)). Thus, the inclusion of a pre-emulsion step aids in the generation of D-W emulsions with higher volume fractions.

The second approach was coupling two reactors in series. The D-W emulsion was generated using two distinct reactors in series configurations. In the first configuration (48 kHz-525 kHz), the decane and aqueous phase were introduced to a WJR operated at 48 kHz and 10 W and the emulsion generated in the first reactor was introduced to a WJR operated at 525 kHz and 10 W. In the second configuration (525 kHz-48 kHz) the reactor order was changed with the first WJR operated at 525 kHz and 10 W followed by the WJR at 48 kHz and 10 W. The continuous and the dispersed phase flow rate were 0.4 ml/min and 0.1 ml/min respectively, which results in residence time of 2 min in each reactor and a total residence time of 4 min.

In the first configuration, the decane slugs are not completely emulsified in the first WJR operated at 48 kHz. The emulsion and the decane slug enter the second reactor where they undergo further emulsification and break up to generate a D-W miniemulsion, as seen in Fig. 14(b). Also, in the second configuration the decane slugs do not undergo complete emulsification in the first WJR at 525 kHz. The D-W emulsion obtained at the outlet of the second configuration has a significantly larger droplet size than the one obtained for the first configuration, as seen in Fig. 14(b). Looking at the droplet size distribution for the 48 kHz-525 kHz configuration, it is apparent that the emulsion and the slugs exiting the first reactor undergo further emulsification and breakup in the second reactor. The emulsion exiting the second reactor has no significant droplet volume size above the linear resonance bubble radius (R_r_ ∼ 5.1 μm) at 525 kHz. For the configuration 525 kHz-48 kHz, the miniemulsion and large droplets exiting the first reactor undergo further breakup in the next reactor operated at 48 kHz. As stated earlier, the droplets will undergo emulsification if they are larger than the cavitation bubble size, which is higher for the frequency of 48 kHz (R_r_ ∼ 56 μm). This results in a final droplet size with a significant number of droplets in the size range of 5–50 μm.

The experiments have confirmed the role cavitation bubble size accompanied by the ultrasound frequency plays in the droplet breakup during emulsification in microchannels. The results also reveal that the configuration is also of prime importance when targeting miniemulsion generation when employing microreactors in series. The coupling of a reactor with a micromixer or another reactor in series, aimed to increase the dispersed phase volume fraction in the miniemulsion, is successfully implemented and offers an alternative approach for continuous miniemulsion generation.

## 4 Conclusions

The effect of the surface roughness of the microchannel on the emulsification mechanism and the emulsion droplet size is studied for microchannels with two different surface roughnesses. The two microchannel etching techniques, namely waterjet cutting and lasered etching, employed for the etching of the microchannel result in pits on the channel side wall. The pits and imperfections on the channel wall localize and promote cavitation activity in its vicinity. The size of the pits in the microchannel is instrumental in promoting stable or transient cavitation, which contributes to the O/W emulsification in the microchannel. We have identified the emulsification modes, viz., fine dispersion and large droplets generation. The emulsification modes are influenced by the cavitation bubble size and position relative to the oil–water interface and oil droplets. In addition, we demonstrate that the cavitation bubble size plays an important role in the droplet breakup in the microchannel. The observations of this mechanistic study are in agreement with the droplet size distribution, which reveals the emulsion droplets resulting from fine dispersion and large droplets generation mode. A further look into the WJR at higher resonance frequencies confirms our mechanistic observations at 48 kHz. We see a gradual decrease in droplet size for O/W emulsion for an increase in the frequency. In addition, we reveal an emulsification mechanism at a frequency of 525 kHz due to standing waves in the microchannel, which has only been reported for frequency above 1 MHz in previous studies. Finally, we demonstrate the application of the WJR at a high frequency of 525 kHz for miniemulsion generation. The coupling of the microreactor with a micromixer or another reactor in series was successfully implemented for the miniemulsion generation with volume fractions of 15–20 %. The miniemulsion was achieved at an energy density of 4.8 × 10^9^ J/m^3^, which is an order of magnitude lower than for the microreactor reported previously and of the same or a higher order of magnitude of continuous or batch ultrasonic emulsifications [1], [7], [24], [25], [36].

The mechanistic study and its learnings could prove valuable in the design of scaled-up reactors for application in emulsification. In addition, the rough microchannels have shown to capture cavitation bubbles and promote transient cavitation in the microreactor. Owing to these advantages, the rough microreactors could prove efficient in particle synthesis, solid handling, and possibly sonochemical synthesis.

## CRediT authorship contribution statement

**Aniket Pradip Udepurkar:** Conceptualization, Methodology, Investigation, Writing – original draft. **Christian Clasen:** Supervision, Writing – review & editing. **Simon Kuhn:** Funding acquisition, Supervision, Writing – review & editing.

## Declaration of Competing Interest

The authors declare that they have no known competing financial interests or personal relationships that could have appeared to influence the work reported in this paper.
