# Supplementary material for: Emulsification mechanism in an ultrasonic microreactor: Influence of surface roughness and ultrasound frequency
Source: Ultrason Sonochem. 2023 Feb 8;94:106323. doi: 10.1016/j.ultsonch.2023.106323 (PMC9945801; doi:10.1016/j.ultsonch.2023.106323)
Supplement: Supplementary data 15 [file mmc15.pdf]

Supplementary Information

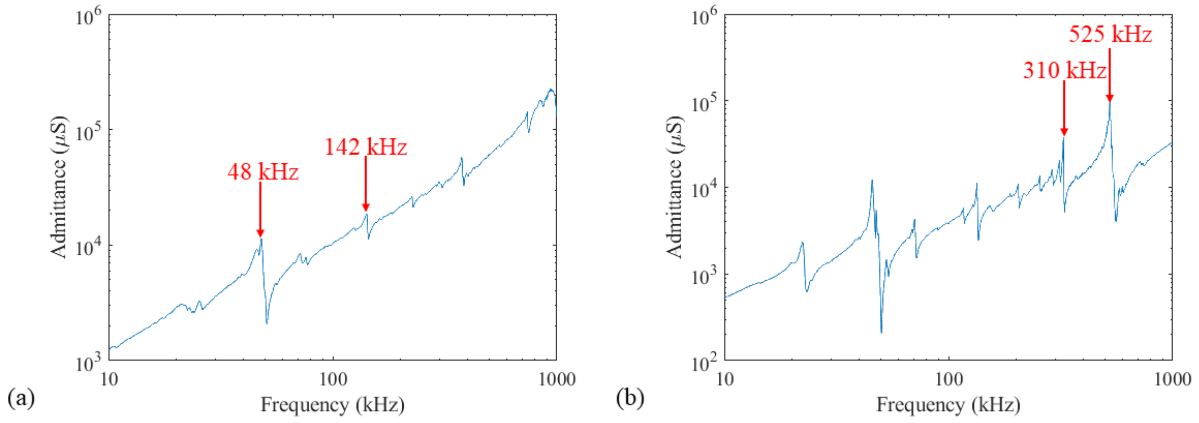

Figure S1: The admittance curve for the borosilicate glass microreactor coupled with a piezoelectric plate transducer of thickness (a) 1.67 mm and (b) 4 mm. The resonance frequencies which are also the operating frequencies for the reactors are stated in the admittance curve.

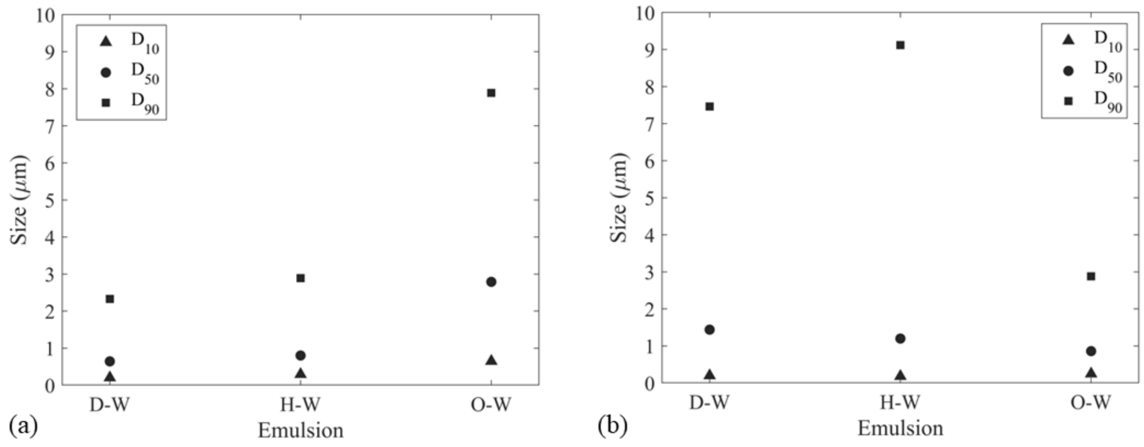

Figure S2: D_{10}, D_{50}, and D_{90} for the droplet size distribution of the decane-in-water (D-W), hexadecane-in-water (H-W), and sunflower oil-in-water (O-W) emulsion generated at the frequency of 48 kHz and power of 20 W. The continuous phase and the dispersed phase flow rate is 0.2 ml/min and 0.05 ml/min respectively and the residence time is 4 mins. The emulsion is generated in (a) Water jet cut microreactor (WJR) and (b) Laser cut microreactor (LCR).

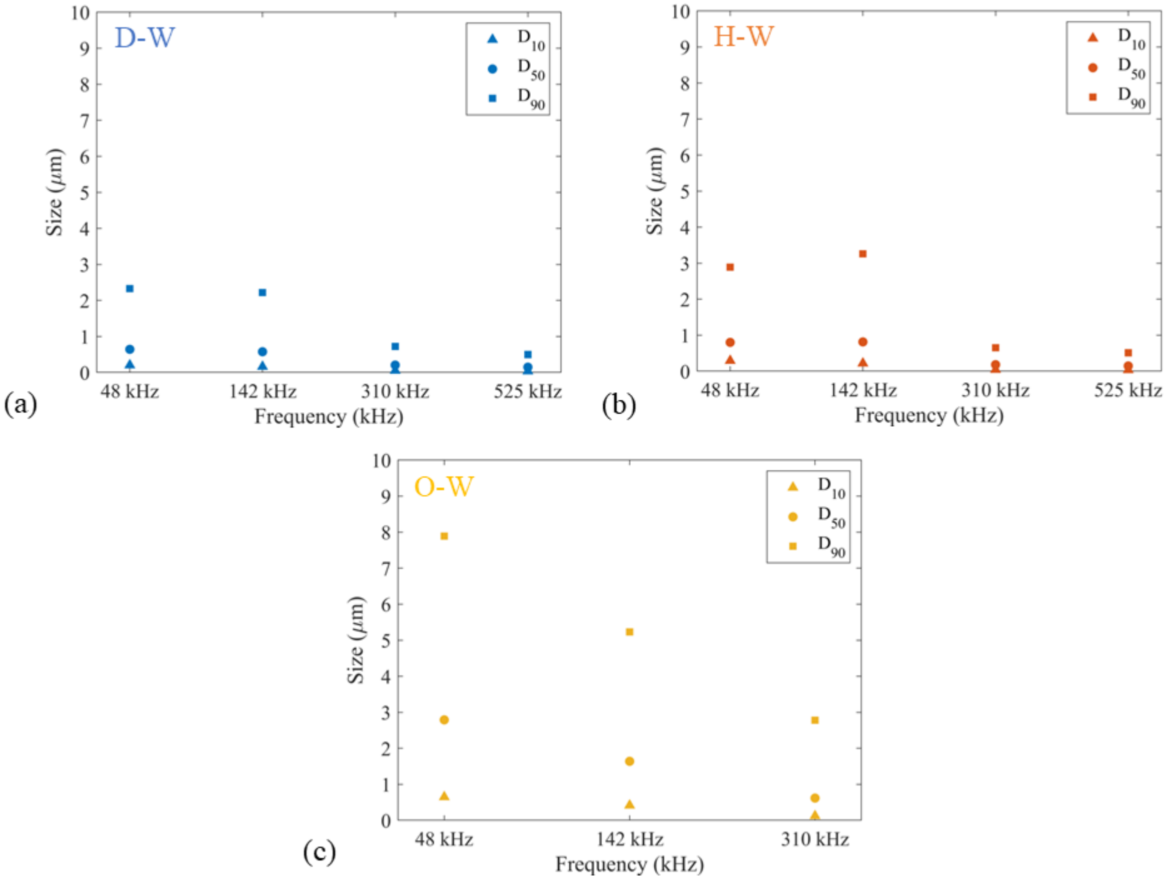

Figure S3: D_{10}, D_{50}, and D_{90} for the droplet size distribution of the decane-in-water (D-W), hexadecane-in-water (H-W), and sunflower oil-in-water (O-W) emulsion generated at power of 20 W at the different resonance frequencies of WJR. The continuous phase and the dispersed phase flow rate is 0.2 ml/min and 0.05 ml/min respectively and the residence time is 4 mins. No significant emulsification was observed for sunflower oil at 525 kHz and thus is not included in (c). The D-W and H-W emulsification was not complete for 20 vol% at 525 kHz, but the droplet size distribution did not change significantly compared to the lower volume fractions where complete emulsification was achieved.

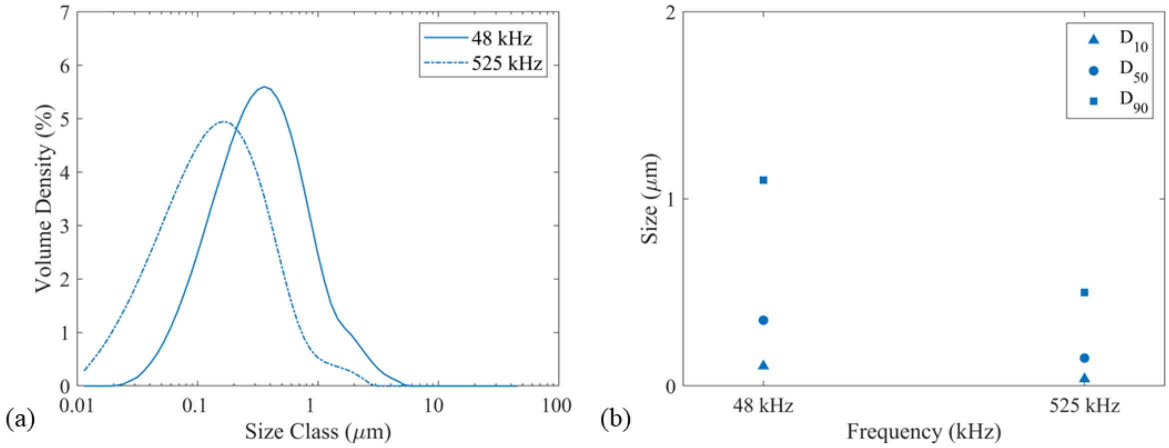

Figure S4: Droplet size distribution and D_{10}, D_{50}, and D_{90} for D-W emulsion generated in a WJR at the frequency of 48 kHz and 525 kHz and power of 20 W. The emulsion with dispersed phase volume fraction of 7.5% is achieved by changing the flow rate ratio of the continuous to the dispersed phase for the total flow rate of 0.25 ml/min and residence time of 4 mins. The continuous and the dispersed phase flow rate are 0.23125 ml/min and 0.01875 ml/min respectively.

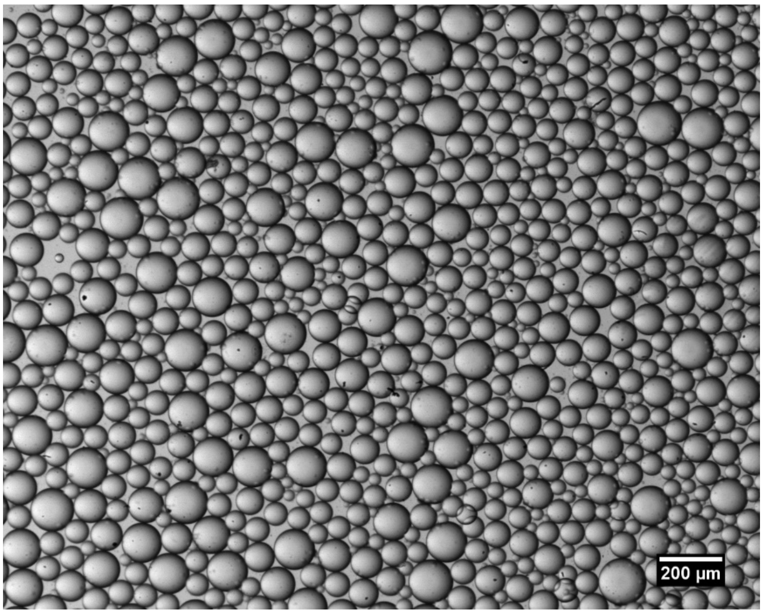

Figure S5: The D-W emulsion generated using a Chemtrix micromixer. The D-W emulsion is collected at the micromixer outlet and a 20 μl emulsion droplet is observed under the microscope. The dispersed phase volume fraction is 17%. The continuous and the dispersed phase flow rate is 0.2075 ml/min and 0.0425 ml/min respectively.

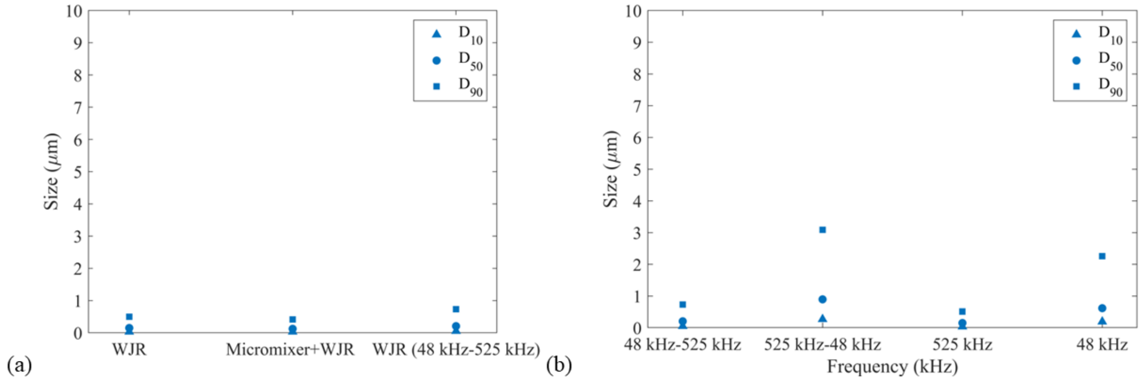

Figure S6: D_{10}, D_{50}, and D_{90} for the droplet size distribution of (D-W) emulsion generated at power of 20 W and frequency of 525 kHz employing an WJR coupled with either a WJR or a micromixer in series. (a) D-W miniemulsion for the WJR (7.5%), Micromixer and WJR in series (17%), and two WJR in series (20%). The decane volume fraction is indicated in the closed brackets. The residence time in the WJR is 4 mins. (b) D-W miniemulsion for two WJR in series in 2 different configurations in comparison with the WJR operated at a single frequency. The first configuration is 48 kHz WJR followed by 525 kHz WJR, and the second configuration is 525 kHz WJR followed by 48 kHz WJR. The single WJR are operated at a total flow rate of 0.25 ml/min, a power of 20W and a decane volume fraction of 20%. The WJR in series are operated at a total flow rate of 0.5 ml/min, a power of 10W for each reactor and a decane volume fraction of 20%. The total residence time is 4 mins in each case.
